# Comprehensive in vivo identification of the c-Myc mRNA protein interactome using HyPR-MS

**DOI:** 10.1261/rna.072157.119

**Published:** 2019-10

**Authors:** Michele Spiniello, Maisie I. Steinbrink, Anthony J. Cesnik, Rachel M. Miller, Mark Scalf, Michael R. Shortreed, Lloyd M. Smith

**Affiliations:** 1 Department of Chemistry, University of Wisconsin–Madison, Madison, Wisconsin 53706, USA; 2 Department of Medicine of Precision, University of Studi della Campania Luigi Vanvitelli, Naples 80138, Italy; 3 Division of Immuno-Hematology and Transfusion Medicine, Cardarelli Hospital, Naples 80131, Italy

**Keywords:** c-Myc, interactomics, proteomics, RNA-binding proteins

## Abstract

Proteins bind mRNA through their entire life cycle from transcription to degradation. We analyzed c-Myc mRNA protein interactors in vivo using the HyPR-MS method to capture the crosslinked mRNA by hybridization and then analyzed the bound proteins using mass spectrometry proteomics. Using HyPR-MS, 229 c-Myc mRNA-binding proteins were identified, confirming previously proposed interactors, suggesting new interactors, and providing information related to the roles and pathways known to involve c-Myc. We performed structural and functional analysis of these proteins and validated our findings with a combination of RIP-qPCR experiments, in vitro results released in past studies, publicly available RIP- and eCLIP-seq data, and results from software tools for predicting RNA–protein interactions.

## INTRODUCTION

Interactions between RNA-binding proteins (RBPs) and mRNAs are crucial for mRNA biology (Glisovic et al. 2008). mRNA molecules are associated with RBPs in mRNA ribonucleoprotein complexes (mRNPs) for their entire life-cycle, from transcription to degradation. The protein content of the mRNPs is subject to dynamic spatiotemporal remodeling and governs mRNA maturation, nuclear export, stability, localization, storage, translation, and degradation (Abdelmohsen and Gorospe 2015; Singh et al. 2015; Coppin et al. 2018). Alteration of the expression, sequence, posttranslational modifications, or binding cofactors of either an RBP or its mRNA regulatory sequence can disrupt normal interactions resulting in the formation of different mRNA splice forms or variation in translation rates, both of which have been linked to neurological, metabolic, and tumoral diseases (Audic and Hartley 2004; Kechavarzi and Janga 2014; Abdelmohsen and Gorospe 2015; Corbett 2018). Consequently, in-depth characterization of specific mRNA-bound proteomes is essential to understanding mRNA biology in physiological and pathological conditions and to identifying new therapeutic targets.

*c-Myc* is one of the most studied oncogenes. It produces a transcription factor that regulates the expression of many genes necessary for ribosomal and mitochondrial biogenesis, glucose, glutamine and lipid metabolism, cell cycle progression, differentiation, and apoptosis (Langa et al. 2001; Dang 2013; Kalkat et al. 2017). Given its broad functions, *c-Myc* gene expression is closely regulated at both transcriptional and posttranscriptional levels, and its protein activity is controlled via posttranslational modifications (Kalkat et al. 2017). c-Myc is therefore vulnerable to dysregulation through gene amplification, chromosome translocation, viral insertion, mRNA and protein destabilization, and variations in protein expression levels, each of which contribute to its involvement in more than half of human cancers (Bernasconi et al. 2000; Gabay et al. 2014; Kalkat et al. 2017). Activation of c-Myc can initiate and maintain many human tumors through the up-regulation of cellular growth mechanisms (Gabay et al. 2014). Thus, it is not surprising that periods of brief c-Myc suppression have been associated with tumor regression in different cancers. Many therapeutic strategies have been evaluated to achieve c-Myc inactivation by decreasing its expression or impairing its functionality at the gene, mRNA, or protein levels (Li et al. 2014).

Numerous publications indicate the importance of posttranscriptional events, such as nuclear export, mRNA stability, translation, and degradation on the regulation of c-Myc expression through interactions between specific RBPs and c-Myc mRNA. For example, c-Myc mRNA nuclear export to the cytoplasm is regulated by the translation initiation factor eIF4E (Tansey 2014). c-Myc mRNA stability can be variably regulated by ELAV1 (also named HuR), a member of the ELAV/Hu (embryonic lethal abnormal vision drosophila-like/Hu antigen) family, depending on specific cell types and conditions (Keene 2007; van Kouwenhove et al. 2011; Simone and Keene 2013). Additionally, the synergistic actions of IGF2BP1 and four associated proteins (HNRNPU, SYNCRIP, YBX1, and DHX9) provide stability to c-Myc mRNA at the coding region determinant (CRD) by restricting mRNA degradation processes (Weidensdorfer et al. 2009).

Regulation of c-Myc mRNA with specific RBPs can also occur at the translational level. CELF1 binding at the 3′-UTR negatively regulates c-Myc translation by preventing c-Myc mRNA from associating with ELAVL1 (Liu et al. 2015). Furthermore, two of the four *c-Myc* promoters, P1 and P2, produce transcripts with long 5′ UTRs containing internal ribosomal entry sequences (IRESs). Proteins including hnRNPC, hnRNPK, PCBP1, PCBP2, hnRNPA1, and RPS25 are all able to modulate c-Myc mRNA translation by interacting with these IRESs (Evans et al. 2003; Kim et al. 2003; Audic and Hartley 2004; Shi et al. 2016). Alternatively, c-Myc mRNA degradation can be mediated by the cooperative action of two ribosomal proteins, RPL5 and RPL11, at its 3′-UTR through recruitment of the RNA-induced silencing complex (miR-24/RISC complex) (Liao et al. 2014); a similar mechanism has been shown for RPS14 via a miR-145/RISC mediated pathway (Zhou et al. 2013). Its degradation can also be mediated by endonuclease activity of APE1 (Barnes et al. 2009). Finally, recent studies show that c-Myc mRNA degradation involves the attachment of CPEB to the cis element in the 3′-UTR of the c-Myc transcript and its interaction with the TOB-CAF1 deadenylation complex (Ogami et al. 2014; Jolles et al. 2018).

These examples highlight the biological importance of the interactions between RBPs and c-Myc mRNA, suggesting this interface as a new target in cancer therapy (Koh et al. 2016). Such treatments have already been attempted with IRES inhibitors; normal interactions with RBPs are blocked, decreasing the rate of c-Myc translation and consequently reducing tumor survival in multiple myeloma, breast, and colorectal cancer models (Vaklavas et al. 2015; Wiegering et al. 2015; Shi et al. 2016).

Although studies have been able to reveal the interactions outlined above, technical limitations have prevented a comprehensive in vivo study of the c-Myc mRNA interacting proteome (Rissland 2017). Despite methods that have allowed capture of the polyadenylated mRNA-bound proteome (Ryder 2016) and MALAT1, NEAT1, and Xist lncRNA proteomes (West et al. 2014; Chu et al. 2015; McHugh et al. 2015), their high cost, complexity, and laborious nature have limited their widespread use (Marchese et al. 2016). We have developed HyPR-MS (hybridization purification of RNA–protein complexes followed by mass spectrometry) to address these issues by simplifying the capture oligonucleotide (CO) design, decreasing the degree of crosslinking and solubilization, and developing a multiplexed release strategy to allow the study of several RNA targets simultaneously. HyPR-MS has already been used to study the proteomes of unspliced full-length HIV RNA and MALAT1-001, NEAT1, and NORAD lncRNAs (Knoener et al. 2017; Spiniello et al. 2018). However, these applications targeted relatively abundant transcripts (>300 copies per cell) (Spiniello et al. 2018), while most RNAs have less than 50 copies per cell (Hanley et al. 2013). Transcripts with lower abundances are challenging to analyze because it is difficult to capture enough protein to meet mass spectrometer detection limits for complex mixtures, while still using a practical number of cells. Overcoming this challenge will facilitate the widespread adoption of in vivo RNA centric methods.

Here we extend the known lower capture limit of HyPR-MS to enable the study of the interacting proteome of c-Myc mRNA in K562 cells (60 copies per cell as estimated by RT-qPCR, see Materials and Methods). This chronic myelogenous leukemia (CML) system (K562) is particularly interesting given the role of c-Myc in leukemia tumorigenesis and specifically in CML progression (Vaqué et al. 2005; Delgado and León 2010; Albajar et al. 2011).

We identified 229 c-Myc interacting proteins using HyPR-MS. The c-Myc interactome encompasses RBPs with known or potential roles in transcription, maturation, transport, localization, stability, decay, and translation of c-Myc mRNA and folding of the nascent protein. Several technical and analytical parameters support the identified interactome, suggesting the utility of HyPR-MS as a tool to reveal the interactome of other mRNA targets at biologically relevant concentrations.

## RESULTS

### HyPR-MS capture performance

RT-qPCR was used to determine that c-Myc mRNA is present in approximately 60 copies per cell in K562 and small scale HyPR-MS experiments were performed using 5 × 10^5^ cells to assess the necessary parameters for large-scale implementation (Supplemental Fig. S1). After this preliminary phase, three large scale HyPR-MS experiments were performed using 10^8^ cells, each with small aliquots taken to measure capture efficiency, specificity, release efficiency, and fold enrichment as indications of large-scale capture performance. These estimates of HyPR-MS efficacy compare c-Myc levels in the captured sample with three controls: an initial lysate sample prior to addition of COs, a sample containing nonspecific binders captured by a scrambled CO (not complementary to any part of the genome) after the c-Myc release, and a sample containing general polyadenylated mRNAs captured by a poly(dT) CO from a small aliquot of lysate. A brief depiction of the HyPR-MS procedure can be found in Supplemental Figure S2.

**FIGURE 1.**
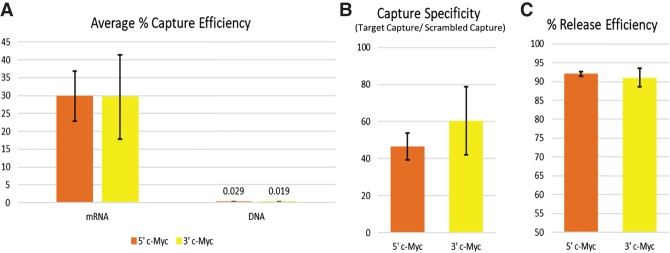
Measures of HyPR-MS large-scale efficacy. (*A*) Average capture efficiencies of all large-scale experiments based on RT-qPCR analysis. (*B*) Average capture specificities in large-scale experiments, comparing the amount of c-Myc target in the capture samples to that in the scrambled capture control samples. (*C*) Average release efficiencies of c-Myc targets from the magnetic beads in large-scale experiments.

Capture efficiency is defined as the percent of mRNA target ultimately eluted from the beads compared to the amount of total target in the initial lysate control. These samples were analyzed using RT-qPCR assays corresponding to regions at the 5′ and 3′ ends of the c-Myc mRNA (Supplemental Fig. S5A), and the capture efficiency calculated based on *C*_t_ values. The capture efficiency of c-Myc in the captured samples averaged 30% for each large-scale experiment (Fig. 1A) mirroring the results of small-scale experiments and confirming that sufficient material had been captured for robust analysis via mass spectrometry (Supplemental Fig. S3A).

The stringency of each large-scale experiment was adjusted to provide high specificity for the captures. Specificity was calculated by comparing the ability of the c-Myc COs to selectively capture target mRNA while minimizing the capture of nontarget mRNAs. The amount of c-Myc in the captured sample was found to be at least 45-fold higher than that in the scrambled control sample for each end of the transcript (Fig. 1B, small-scale Supplemental Fig. S3B). c-Myc mRNA levels were also compared to GAPDH mRNA levels as a second measure of specificity (Supplemental Fig. S4).

The capture and release of oligonucleotides can interact with DNA as well as RNA targets. To ensure DNA-associated proteins did not contaminate the captured material, levels of DNA present in each captured sample were measured with qPCR. As shown in Figure 1A, the capture efficiency for c-Myc DNA is below 0.03%, eliminating the possibility of detecting DNA-associated protein contaminants by mass spectrometry.

Release efficiency indicates the degree of target elution from the beads, which affects both capture efficiency and specificity. Ideally, all targeted mRNA molecules would be released from the beads to achieve maximum capture efficiency, and minimal nonspecific targets would be released to achieve maximal capture specificity. Based on the RT-qPCR results, release efficiencies for 5′ and 3′ c-Myc regions were 92% and 91%, respectively (Fig. 1C, small-scale Supplemental Fig. S3C).

### Characterization of the c-Myc protein interactome

After release, proteins from each c-Myc captured, scrambled, poly(dT), and lysate samples were purified using eFASP (Erde et al. 2014) and digested with trypsin prior to mass spectrometry analysis. Two technical replicates of each biological replicate were analyzed by HPLC-ESI MS/MS. The LC-MS/MS results were normalized using MaxQuant software (version 1.5.3.30) (Cox and Mann 2008), and a finalized list of c-Myc mRNA-enriched proteins was compiled by comparing the captured protein lists with the scrambled, lysate, and poly(dT) controls and removing keratin- and biotin-related contaminants. Based on protein fold-enrichment, 229 proteins were identified as c-Myc interactors (Supplemental Table S1). We used Gene Ontology (GO) Term Enrichment to define each component of the interactome by terms relating to their molecular functions, cellular components, and biological processes (Supplemental Table S2).

**FIGURE 2.**
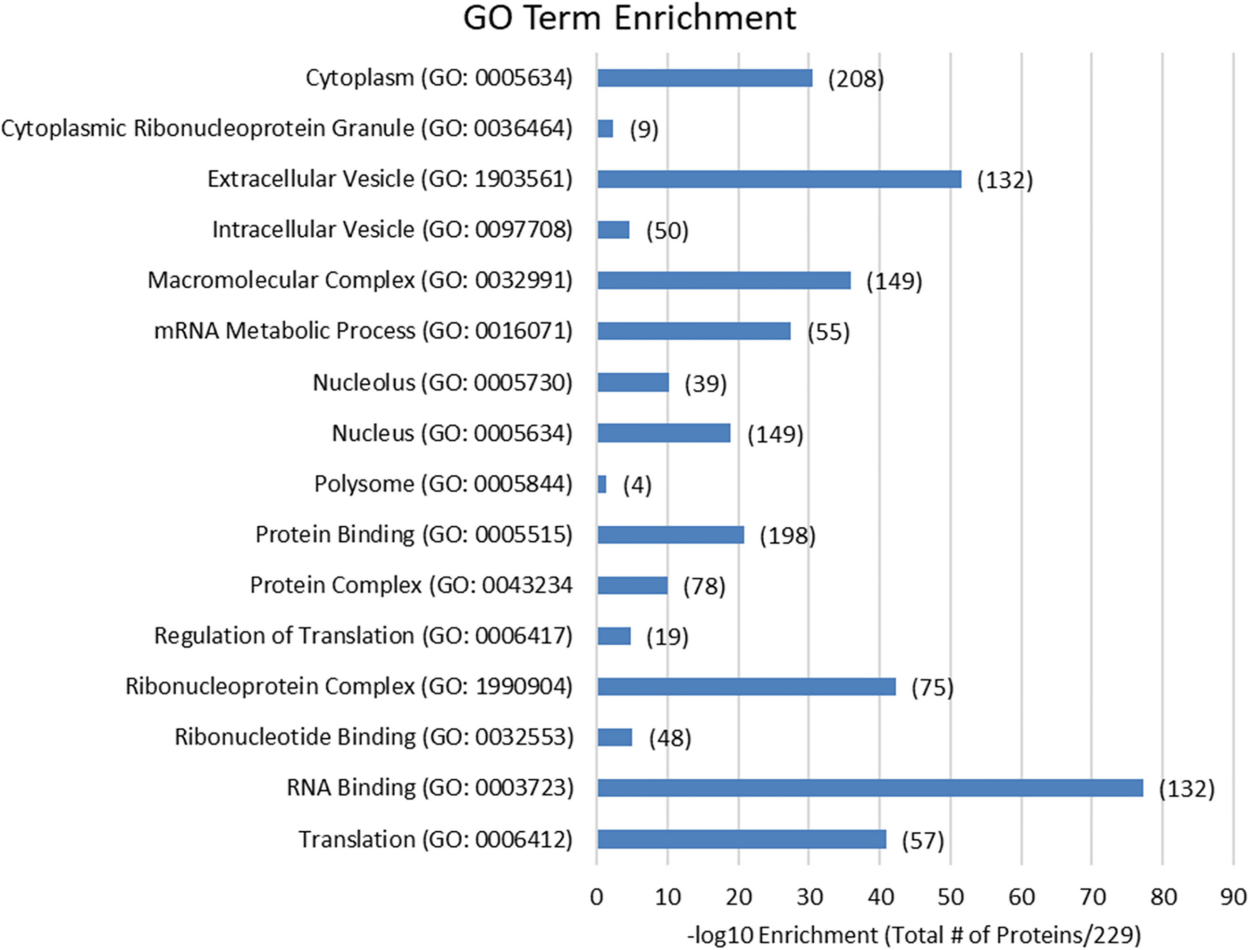
GO term enrichment results. Key GO terms are listed along with −log_10_ enrichment values and the total numbers of proteins with that GO term within the c-Myc enriched list. For all GO term results, see Supplemental Table S2.

The formaldehyde-based crosslinking conditions used in HyPR-MS stabilize both RNA–protein and protein–protein interactions, allowing both direct and indirect protein interactors to be identified (Spiniello et al. 2018). Consequently, we expected to find enrichment of proteins with RNA-binding motifs and proteins that form complexes. GO analysis revealed that more than half of the identified interactors (132 of 229 proteins; *P*-value: 3.17 × 10^−76^) are annotated with the molecular function “RNA-binding,” while 53 proteins (*P*-value: 2.66 × 10^−06^) fall under the term “ribonucleotide binding” (Fig. 2; Supplemental Table S2). Protein-specific information from the UniProt database confirms that 46 c-Myc transcript interactors have documented RNA-binding domains (Supplemental Table S3; Zhang et al. 2010; Jankowsky and Harris 2015). The enrichment of cellular component GO terms, such as “protein-containing complex” (151 proteins; *P*-value: 4.23 × 10^−32^), “ribonucleoprotein complex” (76 proteins; *P*-value: 1.03 × 10^−41^), and “cytoplasmic ribonucleoprotein granule” (12 proteins; *P*-value: 1.49 × 10^−2^) suggests that many c-Myc RBPs interact with each other in mRNPs (Fig. 2; Supplemental Tables S2, S3). In fact, most mRNAs exist as components of mRNPs rather than as naked transcripts because the regulatory nature of these complexes allows the mRNA to fulfill its various posttranscriptional functions (Rissland 2017). Specifically, 65 proteins identified as c-Myc interactors are part of structural RNP complexes, including IGF2BP1-dependent mRNP granules, U1, U11/U12, U7 snRNPs, 40S and 60S ribosomal subunits, RISC, spliceosome C, and tRNA-splicing ligase complexes (Supplemental Table S3).

Five of the c-Myc interacting proteins identified are already well-known c-Myc mRNA interactors: ELAVL1, IGF2BP1, PABPC1, RPL11, and RPS25 (Supplemental Table S3; Keene 2007; Weidensdorfer et al. 2009; van Kouwenhove et al. 2011; Simone and Keene 2013; Liao et al. 2014). Using STRING software (https://string-db.org/) and the BioGRID interaction repository database (https://thebiogrid.org/), we were able to find proteins experimentally determined or predicted to interact with these five known interactors. These proteins represent new potential indirect c-Myc mRNA interactors, consistent with the propensity for RBPs to work together within mRNPs (Supplemental Table S3). Specifically, 36 c-Myc-enriched proteins are known interactors with ELAVL1 (Supplemental Table S4) and may comprise parts of the ELAV/Hu mRNA “regulon,” a dynamic ribonucleoprotein structure postulated to posttranscriptionally regulate multiple functionally related mRNAs simultaneously, including the c-Myc transcript (Keene and Tenenbaum 2002; Keene 2007; Simone and Keene 2013). The GO analysis of these 36 proteins showed enriched GO terms relating to “protein-containing complex” (24 proteins; *P*-value: 4.14 × 10^−4^), “ribonucleoprotein complex” (12 proteins; *P*-value: 1.83 × 10^−5^) and “RNA binding” (22 proteins; *P*-value: 2.30 × 10^−12^) (Supplemental Table S4).

**FIGURE 3.**
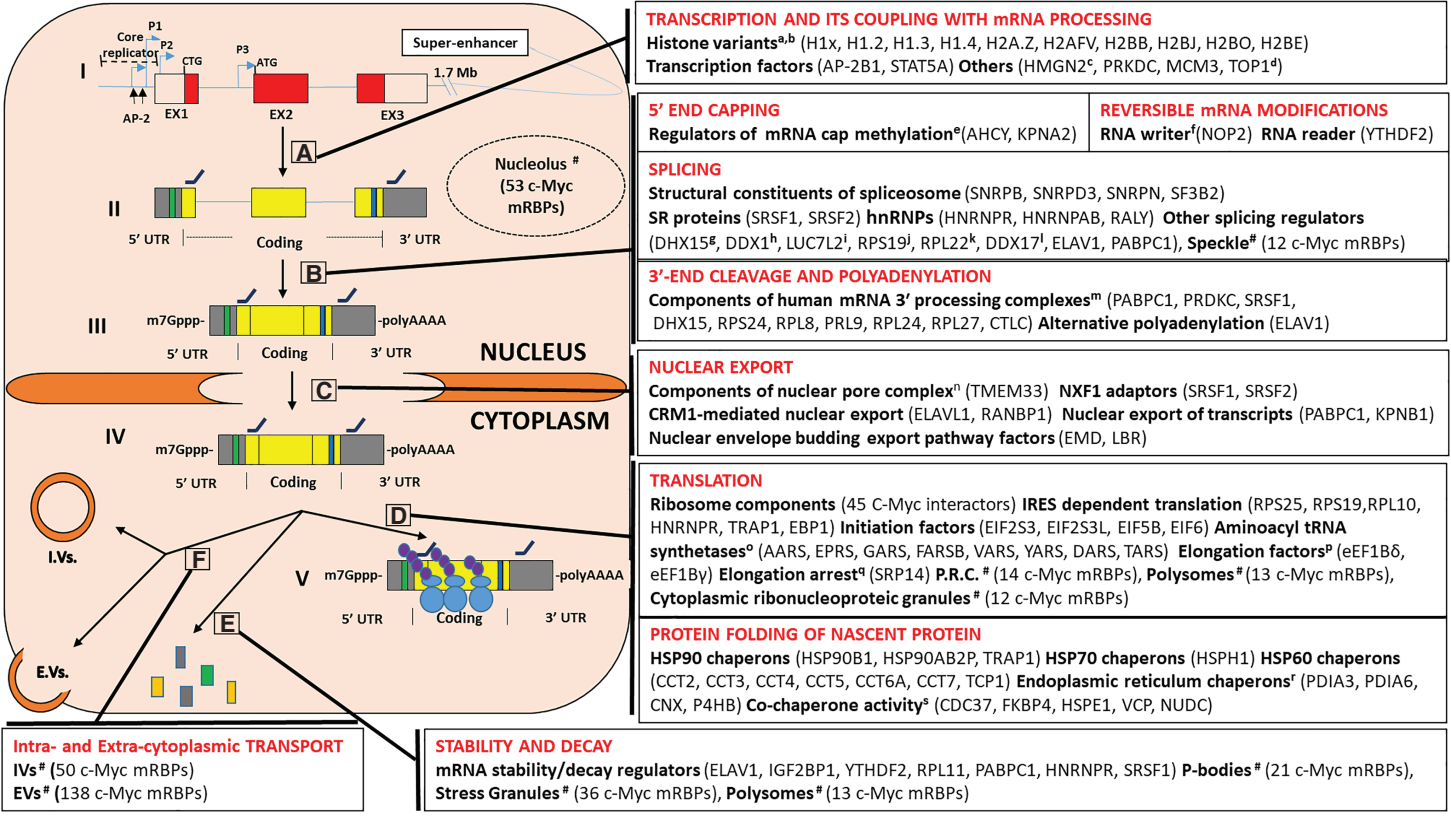
c-Myc mRNA processing and related HyPR-MS identified interactors. c-Myc processing, starting with the gene (I) and the following transcription into pre-mRNA (II), nuclear (III) and cytoplasmic (IV) mature mRNAs, and finally ending with mature mRNA immediately prior to translation. c-Myc RBPs identified using HyPR-MS are listed in boxes corresponding to specific phases of c-Myc mRNA processing, and some occur in multiple boxes if they are involved in several processing roles. (A) Transcription-related c-Myc proteins did not include canonical histones, but histone variants that replace canonical histones can impact processes such as transcription and RNA processing (Tolstorukov et al. 2012; Henikoff and Smith 2015; Buschbeck and Hake 2017; Talbert and Henikoff 2017). (B) Maturation, including 5′ end capping, reversible mRNA modification, splicing and 3′ end cleavage and polyadenylation (C) Nuclear export (D) Translation (P.R.C. = perinuclear region of cytoplasm) and protein folding of the nascent protein (E) Stability and decay (F) Recent findings show vesicle-coupled transport for mRNP complexes in fungi, supporting the hypothesis of similar mechanisms of mRNA transport in higher eukaryotes (Jansen et al. 2014; Haag et al. 2015). In line with this, both IVs and EVs are enriched GO terms by gene ontology analysis (Supplemental Table S3). For proteins not explained in the text we refer to related citations delineating their roles in mRNA processing: ^a^(Harshman et al. 2013), ^b^(Bonenfant et al. 2006), ^c^(González-Romero et al. 2015), ^d^(Naro et al. 2015; Li and Liu 2016), ^e^(Cowling 2009), ^f^(Blanco and Frye 2014; Trixl and Lusser 2018), ^g^(Murakami et al. 2017), ^h^(Zhong et al. 2018), ^i^(Singh et al. 2013), ^j^(Orrù et al. 2007), ^k^(Zhang et al. 2017), ^l^(Dardenne et al. 2014), ^m^(Shi et al. 2009), ^n^(Kabachinski and Schwartz 2015), ^o^(Hausmann et al. 2007), ^p^(Li et al. 2013), ^q^(Zhang and Shan 2012), ^r,s^(Caplan 2003; Chen et al. 2005; Zheng et al. 2011; Saibil 2013; Halperin et al. 2014; Briones 2015; Gao et al. 2016; Narayanan et al. 2016). See Supplemental Table S3 for c-Myc RBPs belonging to these specific subgroups.

Following transcription, mRNAs may aid in maturation, transport and localization, stability maintenance, storage, degradation, and translation through the dynamic spatiotemporal remodeling of the associated mRNP structures (Abdelmohsen and Gorospe 2015; Singh et al. 2015; Coppin et al. 2018). Several c-Myc mRNA interacting proteins have a defined role in different aspects of mRNA processing such as 5′ end capping, reversible mRNA modification, splicing, 3′ end capping and polyadenylation, nuclear export, stability and decay, translation and folding of nascent proteins (Fig. 3). Encouragingly, GO terms related to maturation (“mRNA metabolic process,” *P*-value: 3.16 × 10^−26^), transport (“intracellular vesicle,” *P*-value: 6.79 × 10^−03^; “extracellular vesicle,” *P*-value: 2.20 × 10^−49^), localization (“nucleus,” *P*-value 3.03 × 10^−16^; “cytoplasm,” *P*-value: 2.77 × 10^−27^; “nucleolus,” *P*-value: 1.01 × 10^−09^; “cytoplasmic ribonucleoprotein granule,” *P*-value: 8.66 × 10^−03^; “polysome,” *P*-value: 1.79 × 10^−08^) and translation (“regulation of translation,” *P*-value: 5.84 × 10^−06^; “translation,” *P*-value: 4.75 × 10^−41^) are enriched in the c-Myc list (Fig. 2; Supplemental Table S2), suggesting that HyPR-MS captured elements of the interacting proteome engaged in a variety of functions. Specifically, both nucleus and cytoplasm GO terms components are enriched (Supplemental Table S2), suggesting that the c-Myc mRNAs sample those locations (Pallavicini et al. 1994).

“Nucleolus” is also an enriched GO term with forty c-Myc mRNA interacting proteins (Figs. 2, 3; Supplemental Tables S2, S3). A total of 53 (23.1%) c-Myc RNA-interacting proteins have been previously identified via mass spectrometry as nucleolar constituents (Anderson et al. 2002; Scherl et al. 2002; Boisvert et al. 2011) (Supplemental Table S3), which is consistent with previous studies showing c-Myc mRNA accumulation in the nucleolus (Bond and Wold 1993; Bains et al. 1997; Ideue et al. 2004; Shimizu et al. 2007; Bártová et al. 2008, 2010). Most of these nucleolar components are ribosomal proteins, and five are known components of the 40S and 26 of the 60S ribosomal complexes (Supplemental Table S3; van de Waterbeemd et al. 2018). Other c-Myc mRNA interacting nucleolar proteins are involved in ribosomal biogenesis (TOP1, FBL, EBNA1BP2, NOP56, and NOP58), are known translation factors (EIF6, SRP14) or are chaperones (CCT2, CCT5) (Scherl et al. 2002). This finding supports the hypothesis postulating that the nucleolus is the site of c-Myc mRNA translation (McLeod et al. 2014) and the place where the c-Myc protein is sequestered, modulating its availability for transcriptional activation or repression of target genes (Sanders and Gruppuso 2005).

Fourteen c-Myc interactors are located within the “perinuclear region of the cytoplasm” (Supplemental Table S3; Supplemental Figure 3), although this GO term does not appear among those enriched (Supplemental Table S2). c-Myc mRNA contains a perinuclear cytoplasmic localization signal within the 3′-UTR, which is thought to allow the fast and efficient import of c-Myc protein into the nucleus, where it acts as a transcription factor (Dalgleish et al. 2001). The term “polysome” is significantly enriched, comprising 13 c-Myc interactors (Figs. 2, 3; Supplemental Tables S2, S3) in agreement with the reported localization of c-Myc mRNA into polysomes (Hesketh et al. 1991; Chiluiza et al. 2011). Another enriched GO term, “extracellular vesicle” (EVs) (Figs. 2, 3), includes many c-Myc RBPs (138 of 229 proteins) (Fig. 2; Supplemental Table S2). EVs are important for pathological progression in disease states, especially in primary tumor growth and metastatic evolution, and they are responsible for transferring oncogenic molecules to other cells throughout the body (Sato-Kuwabara et al. 2015; Becker et al. 2016). Lipids, proteins, RNA, and DNA can all be involved in this process, including mRNA transcripts that encode transcription factors (Eirin et al. 2014; Soung et al. 2017); in particular, c-Myc mRNA has been identified in EVs from human medulloblastoma tumor cell lines (Balaj et al. 2014). Within the K562 cell line, EVs can transfer the *BCR/ABL* hybrid gene to normal neutrophils, promoting leukemic cancer progression (Cai et al. 2014). It is likely, given these findings, that c-Myc mRNA could also be present in EVs of K562 cells and promote cancer development.

#### Known c-Myc RBPs identified by HyPR-MS

Five previously noted c-Myc mRNA interactors were identified by HyPR-MS: IGF2BP1, ELAVL1, YTHDF2, RPL11, and RPS25. Specifically, IGF2BP1 together with HNRNPU, SYNCRIP, YBX1, and DHX9 (the latter three of which are present in our c-Myc captured proteins but did not meet the parameters for enrichment) form a coding region instability determinant (CRD)-associated regulatory complex (blue boxes within the transcripts depicted in Fig. 3) to prevent translation-coupled decay of c-Myc mRNA (Weidensdorfer et al. 2009). This stabilization process may occur by IGF2BP recruiting c-Myc transcripts into “protective” mRNPs or stress granules (Bley et al. 2015). ELAV1 can variably regulate c-Myc mRNA stability depending on specific cell types and conditions, either directly by binding AREs within the 3′-UTR of the c-Myc transcript (Fig. 3), or by cooperating with other proteins such as AGO2 or EIF4. (Keene 2007; Kim et al. 2009; Weidensdorfer et al. 2009; van Kouwenhove et al. 2011; Simone and Keene 2013). This is accomplished by alternative polyadenylation and splicing via competition for binding sites (Neve et al. 2017). YTHDF2, a N6-methyladenosine (m6A) “reader” protein, is a confirmed binder of c-Myc mRNA and is involved in increasing stability and expression of c-Myc in K562 cells, the same cells used in this study (Su et al. 2018). The m6A modification can be added to nascent transcripts during maturation events (Ke et al. 2017) and correlates with enhanced c-Myc mRNA translation (Hong 2018). RPL11 is involved in the regulation of c-Myc mRNA stability and promotes degradation by binding to the 3′-UTR and recruiting the RNA-induced silencing complex (miR-24/RISC complex) to the c-Myc transcript in response to ribosomal stress (Challagundla et al. 2011). Finally, RPS25 binds to c-Myc mRNA IRES and stimulates its activity during endoplasmic reticulum stress in multiple myeloma cells (Shi et al. 2016).

#### New c-Myc RBPs identified by HyPR-MS

Among the most interesting new c-Myc RBPs identified (Fig. 3A–D) are: the histone variant H2A.Z; transcription factors (AP-2B1, STAT5A); PRKDC; MCM3; structural constituents of the spliceosome (SNRPB, SNRPD3, SNRPN, SF3B2); proteins with multiple functions in mRNA metabolism such as members of the SR family (SRSF1, SRSF2) and heterogeneous nuclear ribonucleoproteins (hnRNPs) (HNRNPR, HNRNPAB, and RALY); PABPC1; proteins involved in nuclear export mechanisms (RANBP1, KPNB1, EMD, LBR); IRES-mediated translation factors (RPS19, RPL10, TRAP1, EBP1); members of HSP90 (HSP90B1, HSP90AB2P, and TRAP1), HSP70 (HSPH1), HSP60 (CCT2, CCT3, CCT4, CCT5, CCT6A, CCT7, TCP1) and endoplasmic reticulum (PDIA6) chaperone families; and proteins with chaperone or cochaperone activity (VCP).

In accordance with the HyPR-MS findings, previous studies have reported an enrichment of H2A.Z at the c-Myc promoter. This is thought to up-regulate c-Myc transcription, in turn activating H2A.Z transcription in a positive feedback mechanism with tumorigenic consequences (Rangasamy 2010; Monteiro et al. 2014). H2A.Z is overexpressed in melanoma, intrahepatic cholangiocarcinoma, breast cancer, prostate cancer, and bladder cancer (Yang et al. 2018a). Furthermore, recent studies show a primary role for H2A.Z in coordinating transcription elongation and splicing (Santisteban et al. 2011; Nissen et al. 2017).

AP2B1, a member of the AP2 family, acts as either an oncogene or tumor suppressor in various human cancers (Yang et al. 2018b). Two different AP-2 binding sites are present at the c-Myc promoter (Fig. 3; Wierstra and Alves 2008), supporting AP2B1 as a c-Myc mRNA interactor and its potential involvement in c-Myc expression regulation. A second member of the AP2 family, STAT5A, is involved in cell proliferation, survival, and transformation and is a known regulator of c-Myc expression (Wierstra and Alves 2008). The identification of STAT5A as an interacting protein of c-Myc mRNA is supported by several recent studies (Pinz et al. 2016; Kalkat et al. 2017), where it was shown to link to the c-Myc super-enhancer, located 1.7 Mb downstream from its coding region (Fig. 3). During transcription, the super-enhancer can fold via chromatin looping to be in close proximity to the active promoter and nascent mRNA (Lee et al. 2015; Mora et al. 2016).

PRKDC is a serine/threonine-protein kinase involved in DNA double-stranded break repair and cell cycle control, impacting proper chromosome segregation during mitosis (Hsu et al. 2012). It has been suggested that PRKDC inhibition causes a down-regulation of c-Myc mRNA expression, decreasing protein levels and reducing c-Myc-driven proliferation (Zhou et al. 2014). This functional link is supported by the HyPR-MS results.

MCM3 is a component of the prereplication complex involved in eukaryotic genome replication; previous studies have shown it binds the 5′ replicator region of the *c-Myc* gene (Fig. 3; Ghosh et al. 2006). Furthermore, Snyder et al. (2009) have shown that MCM3 colocalizes with RNA polymerase II on the chromatin of transcribed genes, potentially acting with the other MCM proteins to unwind DNA during transcription elongation. The close proximity between the DNA and c-Myc mRNA transcription site during these processes could explain the observed MCM3 interaction with c-Myc mRNA, as cross-linking during HyPR-MS could bind them together.

SNRPB, SNRPD3, SNRPN, and SF3B2 are structural constituents of the spliceosome: (Will and Lührmann 2011). SNRPB and SNRPD3 are Sm family proteins that are closely associated with RNA processing mechanisms and are constituents of critical RNA-associated small nuclear ribonucleoproteins (snRNPs); studies in yeast have shown that both SNRPB and SNRPD3, together with SNRPD1, interact directly with processed pre-mRNA at its 5′ splice site (Zhang et al. 2001). Similarly, SF3B2 is a subunit of the SF3b complex, which binds pre-mRNA at or near a branch site, anchoring the U2 snRNP to the transcript (Cretu et al. 2016).

Members of the SR and hnRNP families are the main mediators of splice site recognition (Busch and Hertel 2012). They play an important role in constitutive and alternative splicing regulation, interacting, respectively, with exonic splicing enhancers and exonic–intronic splicing silencers to recruit spliceosomal components to the pre-mRNA for splice site recognition (Long and Caceres 2009; Busch and Hertel 2012; Chabot and Shkreta 2016). Furthermore, SRSF1 and SRSF2 have been shown to work as adaptors in the Nxf1-driven pathway, used mostly for nuclear export of mRNAs (Delaleau and Borden 2015; Müller-McNicoll et al. 2016). HNRNPR modulates cap-independent translation rates in the cytosol by linking target mRNA IRESs (Geuens et al. 2016). Finally, HNRNPR and SRSF1 have been reported in mRNA stabilization (Lemaire et al. 2002; Sun et al. 2011; Cappelli et al. 2018) and could play this role for c-Myc transcripts as well.

PABPC1 can regulate 3′ mRNA alternative cleavage, polyadenylation and hyperadenylation (Kumar and Glaunsinger 2010; Li et al. 2015). It is also known to bind “stability sequences” such as AREs, poly(A) tails, and CRD that are all present on the c-Myc transcript (Fig. 3; Laird-Offringa et al. 1989; Lemm and Ross 2002; Gorgoni and Gray 2004). Furthermore, PABPC1 can inhibit enzymes during 3′–5′ mRNA decay that use deadenylase activity to remove the poly(A) tail and subsequently degrade the remaining exome (Brewer 1999; Garneau et al. 2007). These are the main players suggested to be involved in Myc transcript degradation. Finally, PABPC1 may interact with c-Myc mRNA during nuclear export, as has been suggested for other transcripts (Mangus et al. 2003).

Several other identified c-Myc mRNA interactors are also associated with nuclear export. RANBP1, along with ELAVL1, acts within the CRM1-mediated pathway to export the mRNAs of proto-oncogenes containing AREs in their 3′-UTR (Köhler and Hurt 2007; Delaleau and Borden 2015), all characteristics exhibited by c-Myc transcripts (Fig. 3). Specifically, ELAV1 binds AREs and associates with CRM1 while RNABP1 permits the release of the mRNA into the cytoplasm (Delaleau and Borden 2015). Although CRM1 is not present in our list, another karyopherin, KPNB1, was identified by HyPR-MS as a c-Myc mRNA interactor and is able to export mRNAs from the nucleus in the cytoplasm (Yi et al. 2002), making it an interesting candidate for involvement in c-Myc mRNA nuclear export. EMD and LBR are two constituents of the inner nuclear membrane proposed to have a role in the nuclear envelope budding pathway (Strambio-De-Castilla 2013), an alternative nuclear export pathway for mRNAs or large mRNPs (Delaleau and Borden 2015). Instances of nuclear envelope budding have been described for herpes virus capsids and mRNP complexes in Drosophila larval body wall muscles; in both cases, protein kinase C (PKC) phosphorylates nuclear lamina, initiating the envelope budding process (Speese et al. 2012). Interestingly, a previous study has shown that PKC inhibitors prevent c-Myc transcript nuclear export, causing nuclear accumulation in peripheral blood mononuclear cells (Selvatici et al. 1998). These studies suggest a potential budding pathway for c-Myc mRNP nuclear export.

RPS19 and RPL10 are ribosomal proteins involved in IRES-mediated translation of mRNAs, affecting erythroid differentiation and BCL-2 transcript, respectively (Horos et al. 2012; Kampen et al. 2019). TRAP1 promotes IRES-dependent translation and attenuates cap-mediated translation; down-regulation of TRAP1 has been associated with lower c-Myc protein levels (Matassa et al. 2014), suggesting it regulates IRES-dependent c-Myc translation. Finally, EBP1 is known to interact with IRES of the foot-and-mouth disease virus, and its expression in K562 is functionally related with cell proliferation, acting on rRNA synthesis and proliferating cell nuclear antigen levels (Monie et al. 2007; Nguyen le et al. 2016). Our finding by HyPR-MS that EBP1 binds c-Myc mRNA suggests EBP1 could promote K562 cell proliferation by regulating c-Myc mRNA expression and specifically its translation initiation. This result and suggested functional importance is in line with previous studies reporting that most of c-Myc translation initiation occurs via the interaction of canonical and IRES-trans-acting factors (ITAFs) with an IRES sequence in the 5′ UTR region of c-Myc transcript (Fig. 3; Stoneley et al. 2000; Cobbold et al. 2010). Only a few initiation factors are present in our list: EIF2S3, EIF2S3L, EIF5B, and EIF6. This makes sense if we consider that several in vitro and in vivo studies show IRES-mediated translation can start with or without a limited set of translation initiation factors, ensuring direct and efficient ribosome recruitment (López-Lastra et al. 2005; Deniz et al. 2009; Johnson et al. 2017).

Members of the HSP90 protein family are known to be involved in the maturation of oncogenic proteins (Chen et al. 2005; Saibil 2013). CDC37, which is able to direct HSP90 proteins to the target protein and modulate their functions, was found to cooperate with c-Myc in tumor transformation in several tissue types (Stepanova et al. 2000; Saibil 2013). HSPH1 is known to bind c-Myc proteins in aggressive B-cell lymphomas, modulating c-Myc expression by preventing its degradation via its chaperone activity. In vitro and in vivo studies show that HSPS1 inhibitors down-regulate c-Myc expression, signiﬁcantly decreasing cell proliferation and tumor growth in B cell lymphomas (Briones 2015). The identification of HSPH1 as a c-Myc mRNA interactor in K562 cells suggests a similar relationship and may indicate the utility to therapeutically target this heat-shock protein in c-Myc driven lymphomas. Seven of the nine members of the HSP60 protein family of chaperonins (Fig. 3D) are found in our list due to their role in c-Myc protein folding (Narayanan et al. 2016; Carr et al. 2017). Finally, PDIA6 and VCP can modulate c-Myc protein levels in bladder and osteosarcoma cancer cells, respectively (Cheng et al. 2017; Heidelberger et al. 2018).

### Confirmation and validation of the HyPR-MS identified c-Myc mRNA protein interactome

The measurements of capture efficiency and specificity, along with release efficiency (Fig. 1) show the technical efficacy of the HyPR-MS method. The number and types of RBPs identified by HyPR-MS also represent an analytical validation of the results: 91.2% (209/229) of proteins are involved in known aspects of mRNA processing, 67.2% (154/229) contain structural motifs characteristic of RBPs and/or mRNPs components, 39.7% (91/229) are defined interactors with known c-Myc mRNA binders, and 6.1% (14/229) are functionally involved in c-Myc transcript or nascent c-Myc protein processing. Supplemental Table S5 lists all the proteins identified in each of these groups and the analytical parameters used to classify them. Only 15 (6.5%) proteins are not present in any of these groups (Supplemental Table S5).

**FIGURE 4.**
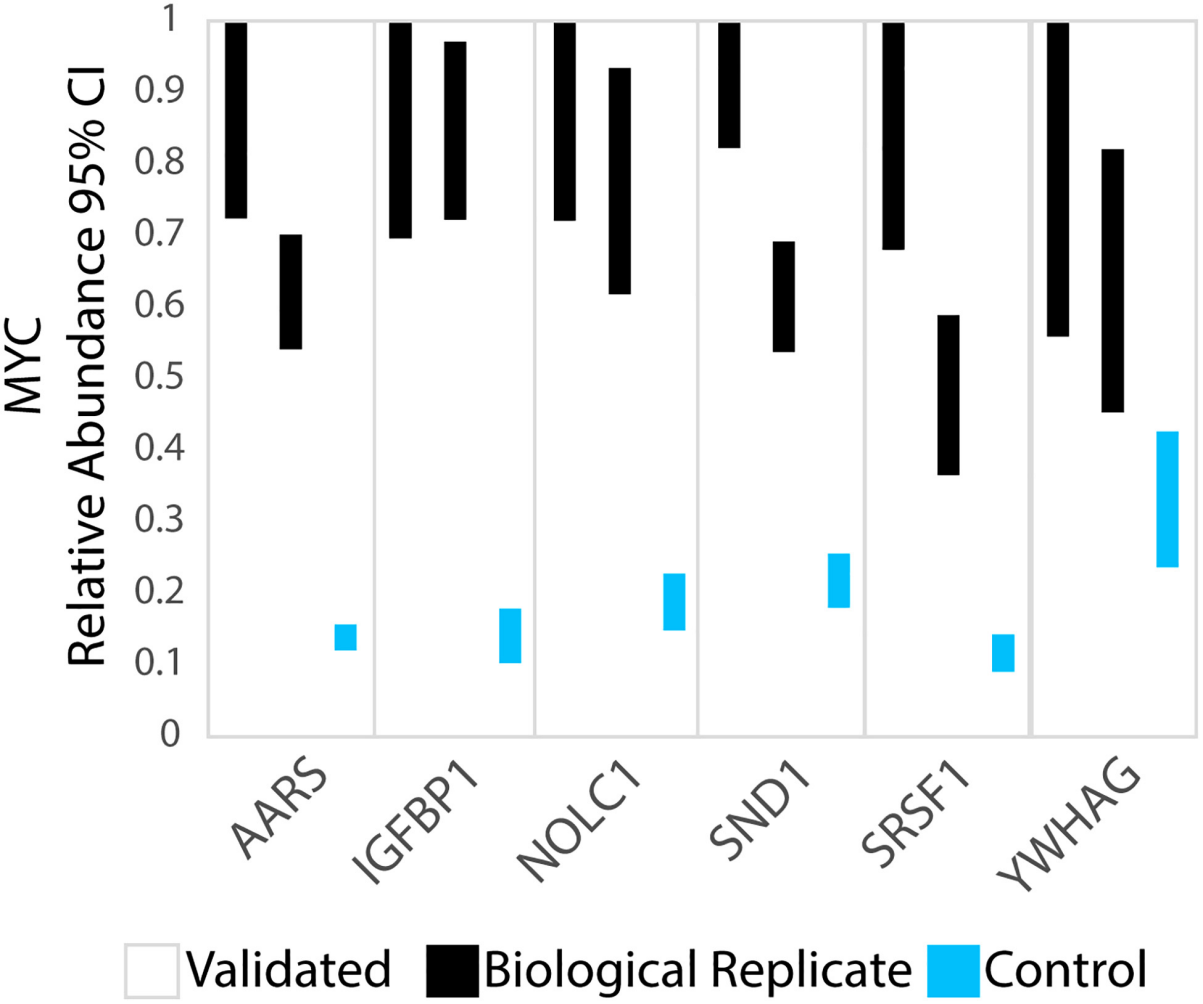
Validation of AARS, IGFBP1, NOLC1, SND1, SRSF1, and YWHAG binding of c-Myc mRNA using publicly available eCLIP data (Supplemental Fig. S6). The 95% credibility intervals for c-Myc mRNA abundances are higher in all eCLIP pull-downs than in the control experiments performed without cross-linking.

We validated 28 of the identified c-Myc mRNA interacting proteins by comparing our data with (a) well defined c-Myc mRNA interactors from published manuscripts (b) potential binders by in vitro methods, (c) predicted partners using dedicated software, (d) publicly available eCLIP-seq data, and finally (e) directly through RIP-qPCR experiments (Fig. 4; Supplemental Table S5). These validation results further support the ability of HyPR-MS to identify real RBPs for a specific target RNA molecule.

#### Literature identification of c-Myc interactors

Through literature analysis, five previously confirmed c-Myc mRNA interactors were identified in our enriched list: ELAVL1, IGF2BP1, YTHDF2, RPS25, and RPL11 (Keene 2007; Weidensdorfer et al. 2009; van Kouwenhove et al. 2011; Simone and Keene 2013; Liao et al. 2014; Su et al. 2018).

#### Literature identification of possible c-Myc interactors

Fifteen HyPR-MS c-Myc RBPs were previously identified as potential c-Myc IRES interactors using in vitro methods. Specifically, PABPC1 and EIF2S3 together with proteins forming the 40S (RPS18, RPS2, RPS9, RPS19, RPS6, RPS20) and 60S (RPLP0, RPL12, RPL13, RPL3, RPL18) ribosomal complexes were identified on a c-Myc IRES construct in native 48S initiation complexes that were subjected to glutathione RNA affinity chromatography purification and mass spectrometry analysis (Thoma et al. 2008). Furthermore, two HyPR-MS-identified proteins, DDX1 and ELAVL1, were previously noted as potential c-Myc mRNA interactors using a protein microarray system (Siprashvili et al. 2012; Marchese et al. 2016).

#### Use of software to predict c-Myc interactors

Recently developed prediction software RBPDB (http://rbpdb.ccbr.utoronto.ca/) and RBPmap (http://rbpmap.technion.ac.il/) are able to predict the presence and location of binding sites between RNA molecules and a selected pool of RNA-binding proteins (Cook et al. 2011; Paz et al. 2014). These programs have been used to determine whether c-Myc mRNA has binding sites for proteins identified as mRNA interactors using HyPR-MS. c-Myc mRNA contains predicted binding sites for several RNA-binding proteins identified here, implying they are direct interactors with exact binding locations and further supporting the utility of HyPR-MS (Supplemental Tables S6, S7). In addition to the previously mentioned ELAVL1 and PABPC1, RBPDB and RBPmap software both detected SRSF1 and SRSF2 as potential interactors. RBPmap additionally predicted RALY to be a c-Myc-associated protein. Each of these proteins is involved in mRNA processing as described previously.

#### Analysis of CLIP-seq data

Publicly available eCLIP-seq data from K562 or HepG2 cells was used to confirm c-Myc mRNA interactors present in our list. In addition to the already cited IGF2BP1 and SRSF1, these results validated AARS, NOLC1, YWHAG, and SND1 as c-Myc-mRNA interactors (Fig. 4).

#### RIP-ChIP validation

**FIGURE 5.**
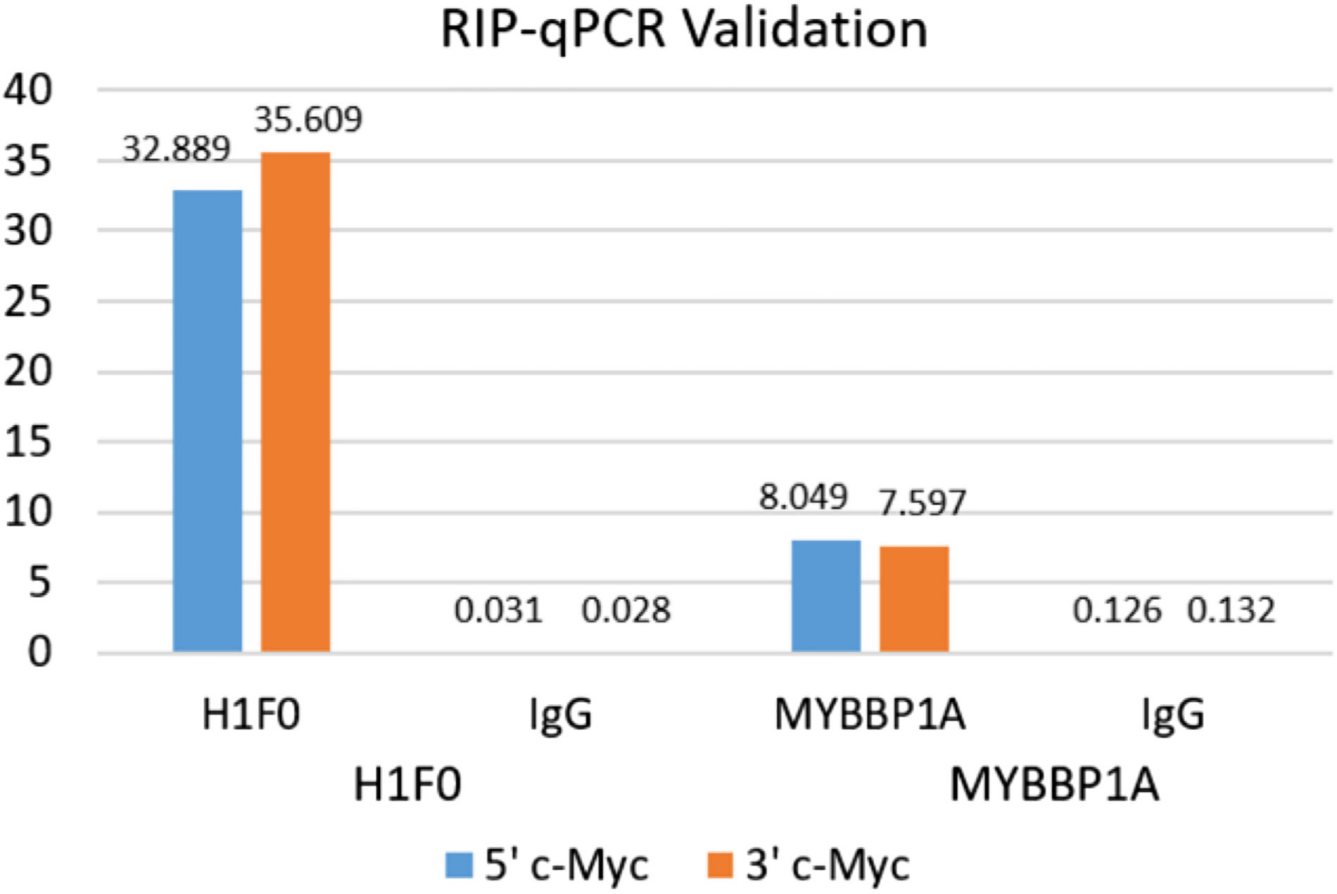
RIP-qPCR validation for H1F0 and MYBBP1A. RIP-qPCR was performed to validate the enrichment of histone variant H1F0 and transcription regulator MYBBP1A with c-Myc in K562 cells. Fold enrichments were calculated using the ΔΔ*C*_t_ method as described in the kit protocol.

Finally, two proteins within the c-Myc enriched list, histone variant H1F0 and Myb-binding protein 1A (MYBBP1A), were experimentally confirmed as c-Myc mRNA interactors by RIP-qPCR analysis compared to an IgG negative control (Fig. 5). Interestingly, an increased expression of H1.X has been found in neuroendocrine tumors (Warneboldt et al. 2008), many of which are driven by c-Myc deregulation (Farrell et al. 2017; Mollaoglu et al. 2017). MYBBP1A, a transcription regulator involved in cell division, cell proliferation and apoptosis (Mori et al. 2012; George et al. 2015), is able to stimulate c-Myc transcription inhibiting c-Myb (Yamauchi et al. 2008). Furthermore, MYBBP1A can bind RNA molecules as the lncRNA PURPL via the adaptor protein HuR, which is also present in our list, forming a complex that regulates p53 protein stability in the nucleoplasm (Li et al. 2017).

## DISCUSSION

To our knowledge, this is the first work that identifies a specific mRNA-bound proteome using an in vivo RNA-centric method. The choice of c-Myc mRNA (60 copies per cell in K562) as the target of our study represents a test of the ability of HyPR-MS to identify the interacting proteomes of RNAs present at biologically relevant concentrations while still using a reasonable (10^8^) number of cells. Furthermore, the regulation of c-Myc expression can happen posttranscriptionally (Sears 2004), and consequently, the comprehensive identification of c-Myc mRNA–protein interactions gives us new insights into the RBPs involved in regulating one of the most important oncogenes, potentially revealing new therapeutic targets.

Using HyPR-MS, we identified 229 c-Myc RBPs, confirming previously proposed interactors, suggesting new interactors, and providing information related to the roles and pathways involving c-Myc. Future functional studies are necessary to test the role of c-Myc mRNA interactors identified here and their impact on *c-Myc* gene expression.

The high capture efficiency and specificity support the efficacy of HyPR-MS pulldowns for the c-Myc transcript. The toehold-mediated release strategy permits copurification of the poly(dT) and scrambled capture probes consecutively with that of c-Myc and thereby allows them to be used as negative controls to assist in data analysis and interpretation. Structural and functional analysis (gene ontology, UniProt, STRING, bioGRID, and previous MS-based studies) confirm the reliability of the identified c-Myc RBPs. Five of these (ELAVL1, IGF2BP1, YTHDF2, RPS25, and RPL11) are known interactors with defined roles in c-Myc mRNA processing. Others were validated by comparing our findings with previous in vitro results, publicly available RIP- and eCLIP-seq data, software predictions, or RIP-qPCR experiments.

Together, these analyses have identified a comprehensive binding proteome for c-Myc mRNA, revealing new insights into its interaction partners. This has uncovered candidates for potential new therapies focusing on one of the most biologically relevant but difficult to drug oncogenes, and demonstrates the power of HyPR-MS to study mRNAs at biologically significant levels.

Future applications of HyPR-MS to the study of mis-spliced transcripts and/or to the comparison between the interacting proteomes of individual RNA targets in physiological and pathological conditions could permit the identification of RNA–protein interactions “ideal” for the development of effective, specific therapies while limiting off-target effects.

## MATERIALS AND METHODS

### Cell culture and growth conditions

K562 cells were cultured in suspension with DMEM medium containing 10% fetal bovine serum (FBS) and 1% penicillin/streptomycin at 37°C and 5% CO_2_. Crosslinking was performed with 1% formaldehyde (w/v) for 10 min at room temperature, then quenched with Tris-HCl (pH 8.0) at a final concentration of 250 mM for 10 min. Cells were washed with cold phosphate buffered saline (PBS) twice, then pelleted by centrifugation at 4°C and flash frozen in liquid nitrogen and stored at −80°C prior to use.

### HyPR-MS method

Lysis of formaldehyde-crosslinked K562 cells, sonication of the resulting lysate, hybridization capture, and bead release were performed as described previously (Spiniello et al. 2018). Briefly, biotinylated c-Myc mRNA, poly(dT) and scrambled capture DNA oligonucleotides (COs) are added to the sonicated lysate and hybridize with their respective RNA targets to create CO–mRNA–protein complexes. After hybridization, streptavidin-modified magnetic beads are added and bind the biotinylated COs to create bead-CO–mRNA–protein complexes. A magnetic Eppendorf rack separates these complexes out of solution so nontarget RNA and proteins can be removed. Following this, release DNA oligonucleotides (ROs) complementary to each CO are sequentially added to specifically elute the target RNA–protein complexes back into solution for collection. Small aliquots can be reverse transcribed and analyzed via RT-qPCR at various stages of the procedure to test measures of method efficiency, while larger samples can be subjected to protein purification and mass spectrometric analysis. All CO and RO sequences and large-scale concentrations are included in Supplemental Information (Supplemental Fig. S5B).

### RT-qPCR

Following HyPR-MS, proteinase K treatment, RNA purification and RT-qPCR were performed on small aliquots of cell lysate, purified target captures, and post-release beads as described in Spiniello et al. (2018) to estimate transcript copy numbers, capture and release effciencies, and capture speciﬁcities. Estimation of c-Myc copy number within the K562 cell line was based on standard curves prepared using genomic DNA (G3041, Promega) using the method described previously (Nolan et al. 2006; Yun et al. 2006). All assay sequences are included in the Supplemental Information (Supplemental Fig. S5C).

### Protein purification and mass spectrometry of peptides

Larger aliquots containing high amounts of c-Myc-, scrambled-, poly(dT)-, and lysate-associated proteins were purified using eFASP and digested with trypsin as described by Spiniello et al. (2018). Briefly, lysate, c-Myc, poly(dT) and scrambled samples were washed through 50 kD centrifugal filters to collect the proteins. These proteins were then washed with an exchange buffer (urea, deoxycholic acid), reducing buffer (urea, dithiothreitol), alkylation buffer (urea, iodoacetamide, ammonium bicarbonate), and digestion buffer (urea, ammonium bicarbonate, deoxycholic acid). Proteins were digested with 1 µg trypsin overnight at 37°C and then eluted from the filter. These solutions were washed with ethyl acetate and TFA before lyophilization via SpeedVac. Each sample was reconstituted in formic acid solution and analyzed in triplicate (three technical replicates for each of the three biological replicates) by HPLC-ESI-MS/MS as performed in Spiniello et al. (2018), except for using ten MS/MS HCD scans of the ten highest intensity parent ions instead of twenty.

### Mass spectrometry data processing

Raw files from the mass spectrometry output for c-Myc mRNA, poly(dT), scrambled and lysate trypsin-digested samples were analyzed using MaxQuant software (http://www.biochem.mpg.de/5111795/maxquant), as described in Spiniello et al. (2018). A protein was ultimately considered to be enriched in the c-Myc mRNA capture samples if its intensity was ≥5-fold greater in the c-Myc mRNA sample than in the scrambled capture sample, lysate, or poly(-dT) sample. Only proteins meeting this criterion in at least two of the three biological replicates were considered enriched for c-Myc transcript capture. Proteins related to keratinization and biotin were removed from this list as likely contaminants. The final lists were then analyzed to confirm already established biological functions and to predict new characteristics for each target.

### Interactome data analysis

Gene Ontology (GO) Enrichment Analysis was performed using the publicly available Gene Ontology Consortium (http://www.geneontology.org/) (Ashburner et al. 2000; The Gene Ontology Consortium 2019). The enriched proteome list associated with c-Myc mRNA was used as input for GO Enrichment Analysis (PANTHER Overrepresentation Test released 12/5/2017; GO Ontology database released 12/27/2017; Reference all genes in Homo sapiens database; FISHER test type) to search for GO terms related to biological process, molecular function, and cellular component.

Protein relationships were explored using the multiple protein search feature in the STRING (https://string-db.org/) (Szklarczyk et al. 2017) and BioGRID (https://thebiogrid.org/) (Oughtred et al. 2019) databases and protein domains were identified using the UniProt database (https://www.uniprot.org/) (The UniProt Consortium 2019).

### RIP-ChIP validation

RIP-ChIP was used to validate the interactions of c-Myc mRNA with MYBBP1A and H1F0 proteins. Antibodies for each were purchased (Anti-MYBBP1A: Novus Biologicals NB100-61050; Anti-H1x: Bethyl Laboratories A304-603A) and used in conjunction with the EZ-Magna Nuclear RIP (Cross-Linked) Nuclear RNA-Binding Protein Immunoprecipitation Kit (EDM Millipore Cat. No. 17-10521). Normal rabbit IgG was purchased through EDM Millipore (Cat. No. 12-370). K562 cells were crosslinked with 1% formaldehyde for 10 min, lysed, and sonicated. Immunoprecipitation, elution, and RNA purification were performed as indicated by the EZ-Magna kit protocol, and DNase I digestion was carried out after these steps (optional DNase I treatment of the sheared cross-linked chromatin was excluded). RT-qPCR was performed as described above, and the ΔΔ*C*_t_ method was used for data analysis as detailed at the end of the kit protocol to calculate fold enrichment above IgG. Fold enrichments exceeding two were considered significant.

### Validation of Myc-binding proteins using eCLIP data

Several binding proteins for c-Myc mRNA were validated using eCLIP immunoprecipitation data from the ENCODE project in either K562 or HepG2 against each binding protein (The ENCODE Project Consortium 2012). The RNAs captured in these pull-downs were quantified using the integrated software solution Spritz (https://github.com/smith-chem-wisc/Spritz) using the “quantify” command, which quantifies genes and transcripts using RSEM (Li and Dewey 2011). First, Spritz prepares reference indices for the STAR (Dobin et al. 2013) alignment software and RSEM quantification software using the human reference genome (GRCh38.81). Then, it downloads FASTQ files from Sequence Read Archive accessions, if available, and calculates gene abundances using RSEM. The parameters used for the “rsem-calculate-expression” program within RSEM were “–time –calc-ci –star –num-threads 24 –output-genome-bam –paired-end,” and this analysis was performed on an Intel Xeon CPU with 24 cores and 128 GB of RAM. These computations took about 9 h: 1 h for aligning reads, 2 h for estimating expression levels, and 6 h for calculating the credibility intervals of the expression levels.

## SUPPLEMENTAL MATERIAL

Supplemental material is available for this article.

## Supplementary Material

Supplemental Material
