# Supplementary material for: Comprehensive in vivo identification of the c-Myc mRNA protein interactome using HyPR-MS
Source: RNA. 2019 Oct;25(10):1337–52. doi: 10.1261/rna.072157.119 (PMC6800478; doi:10.1261/rna.072157.119)
Supplement: Supplemental Material [file supp_25_10_1337__index.html]

Comprehensive in vivo identification of the c-Myc mRNA protein interactome using HyPR-MS — Supplemental Material 

# Comprehensive in vivo identification of the c-Myc mRNA protein interactome using HyPR-MS

## Supplemental Material

- Supplemental\_Figures\_.docx
- Supplemental\_Table\_S1.xlsx
- Supplemental\_Table\_S2.xlsx
- Supplemental\_Table\_S3.xlsx
- Supplemental\_Table\_S4.xlsx
- Supplemental\_Table\_S5.xlsx
- Supplemental\_Table\_S6.xlsx
- Supplemental\_Table\_S7.xlsx
