# Supplementary material for: Comprehensive in vivo identification of the c-Myc mRNA protein interactome using HyPR-MS
Source: RNA. 2019 Oct;25(10):1337–52. doi: 10.1261/rna.072157.119 (PMC6800478; doi:10.1261/rna.072157.119)
Supplement: Supplemental Material [file supp_072157.119_Supplemental_Figures_.docx]

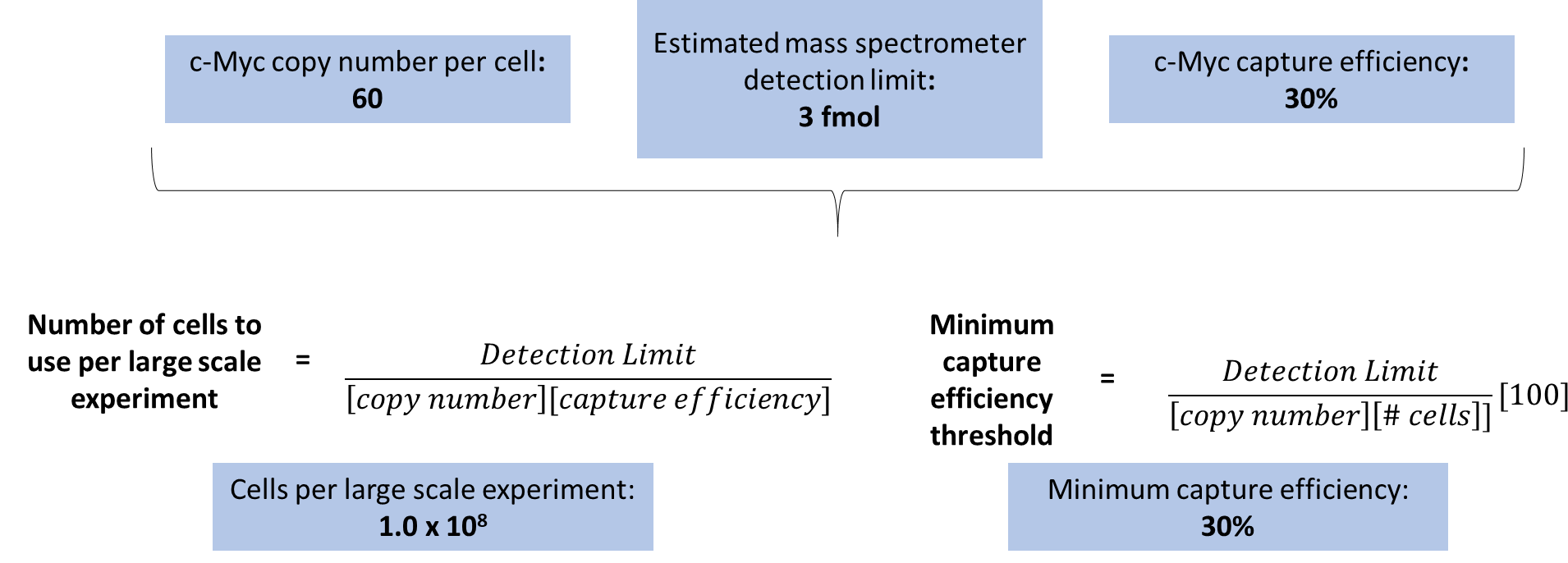


**A**

Figure S1: Determination of Large-Scale Parameters.

A) Based on several preliminary small-scale experiments, the average c-Myc copy number per cell and c-Myc capture efficiency was determined. Along with knowledge of the mass spectrometer detection limit for complex biological samples, these values were used to calculate important parameters for each large-scale experiment. Specifically, we calculated the number of cells to use and the minimum capture efficiency that would be necessary with that number of cells to get reliable mass spectrometry results for protein interactors.


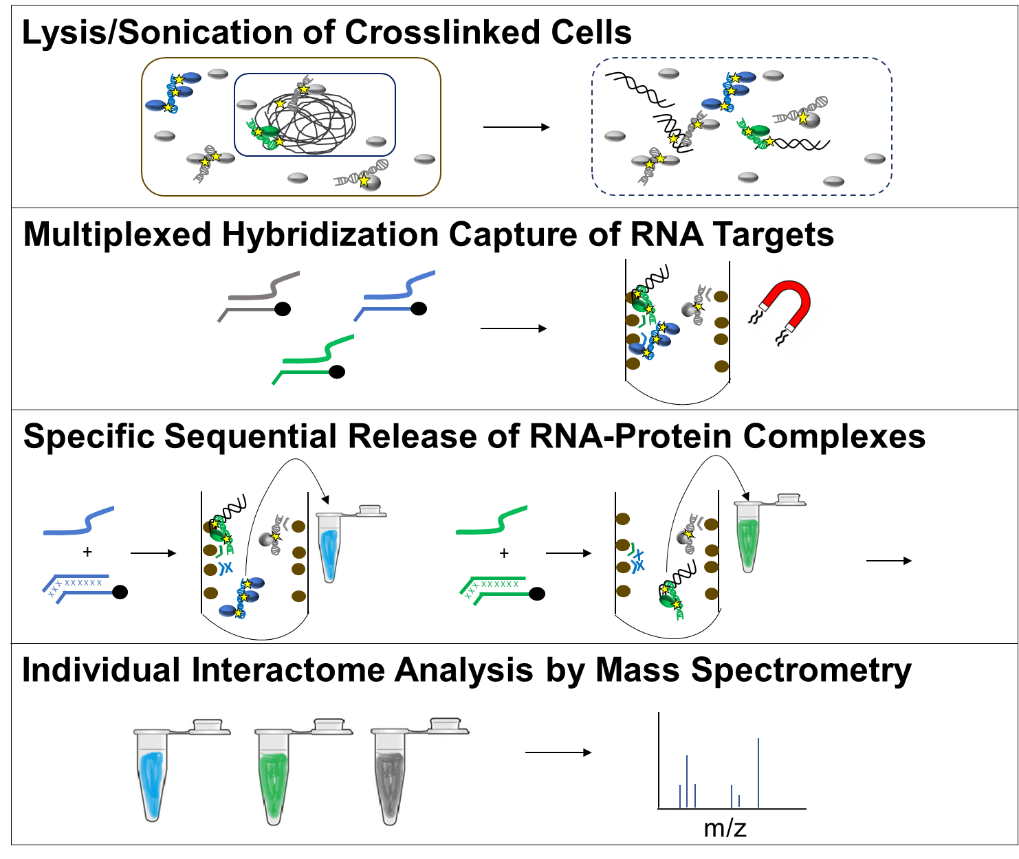

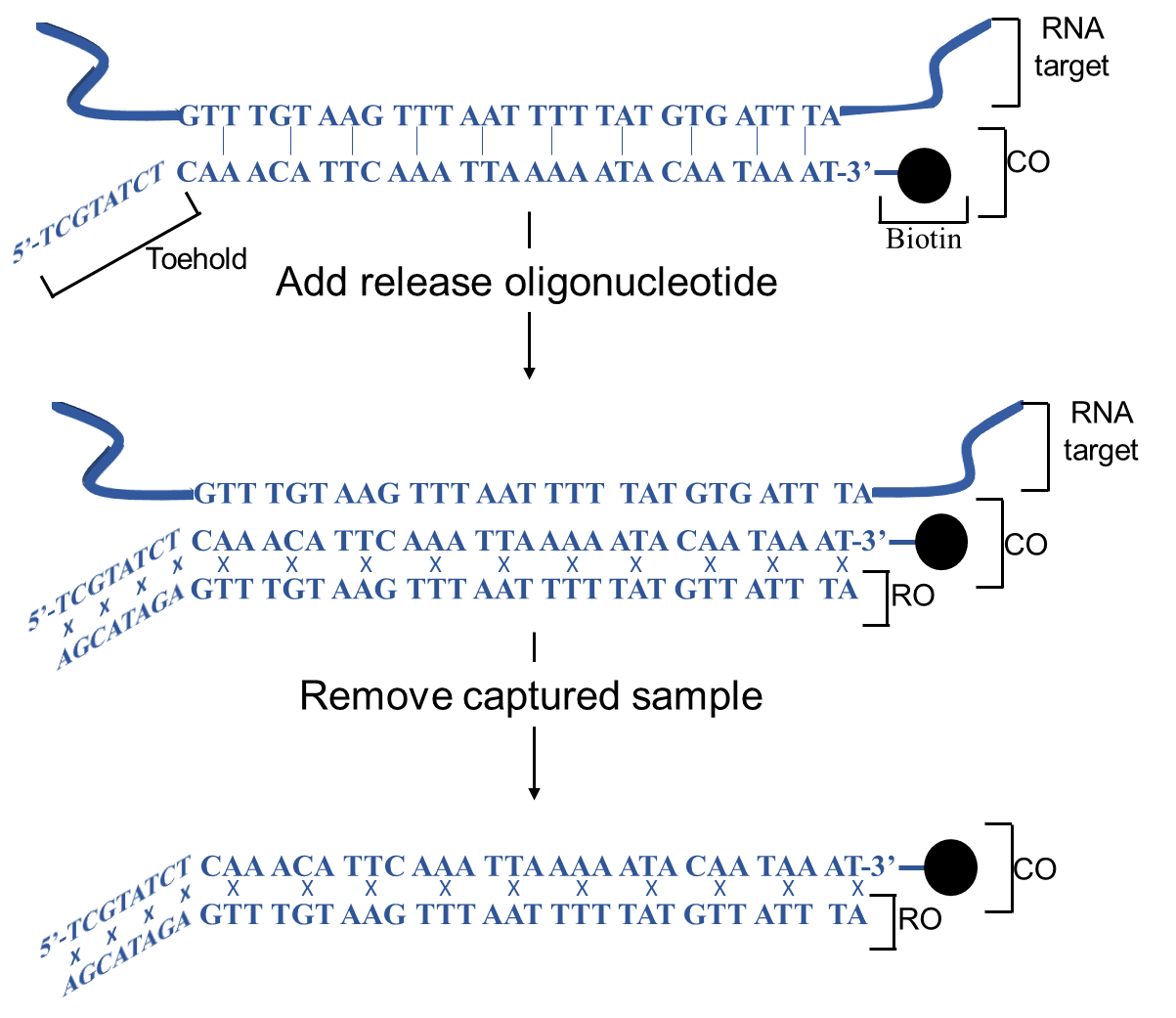


**B**

**A**

Figure S2: HyPR-MS Method.

A) HyPR-MS combines targeted capture of multiple RNA targets with sequential release to allow for multiple interactomes to be analyzed within a single lysate sample. Biotinylated capture oligonucleotides (COs) complementary to a number of desired RNA sequences are added to formaldehyde-crosslinked cell lysate and hybridize to the targets. Streptavidin-coated magnetic beads bind to the biotin modifications on the COs, pulling them, the RNA targets, and the crosslinked proteins out of solution. Excess unwanted cellular material is removed, and the bead complexes are resuspended in solution containing a release oligonucleotide (RO) that is complementary to the full length of one CO. This displaces one RNA target, releasing it into solution to be collected. This sequential release can be performed for several RNA targets. The isolated release solutions are then prepared for mass spectrometry analysis.

B) The release strategy of HyPR-MS is dependent on a toehold-mediated capture. COs are complementary to 25-30 nucleotides (nt) of the target RNA but also contain an 8 nt toehold sequence that does not hybridize to the target. ROs are designed to be complementary to the entire CO sequence (including the toehold), giving RO-CO hybridization higher thermodynamic stability and thus releasing the RNA target into solution.


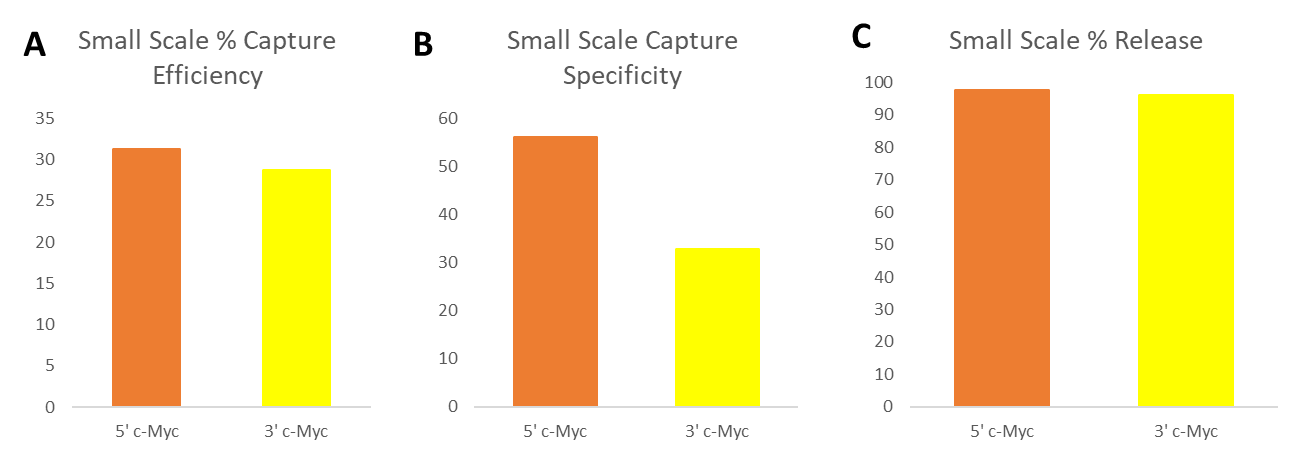


Figure S3: Measures of HyPR-MS Small-Scale Efficacy.

A-C) Small-scale experiments were performed to determine the best sequences and concentrations for COs, ROs, and beads. The conditions yielding the best combination of capture efficiency, specificity, and percent release, shown above, were scaled up to perform large-scale experiments.


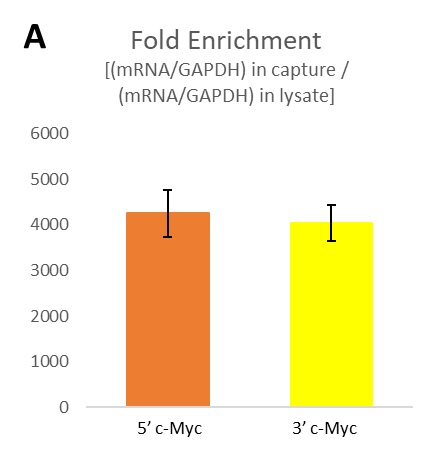


Figure S4: Additional Measures of HyPR-MS Specificity.

A) In addition to capture specificity, the fold enrichment of c-Myc mRNA over GAPDH was determined. To do this, the ratio of target-to-GAPDH in the captured sample was compared to the ratio of target-to-GAPDH in the lysate via RT-qPCR analysis. The fold enrichment of c-Myc was approximately 4,000-fold for each end of the c-Myc transcript.


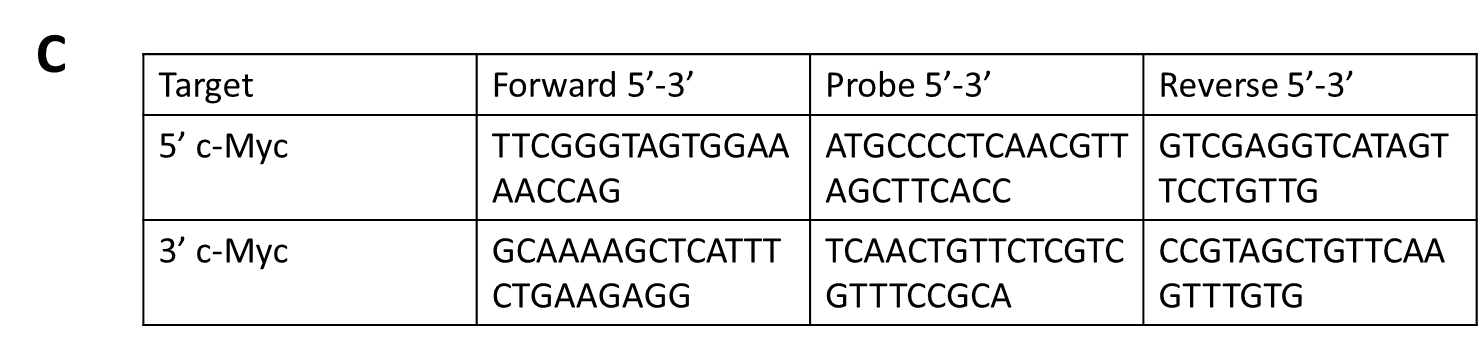

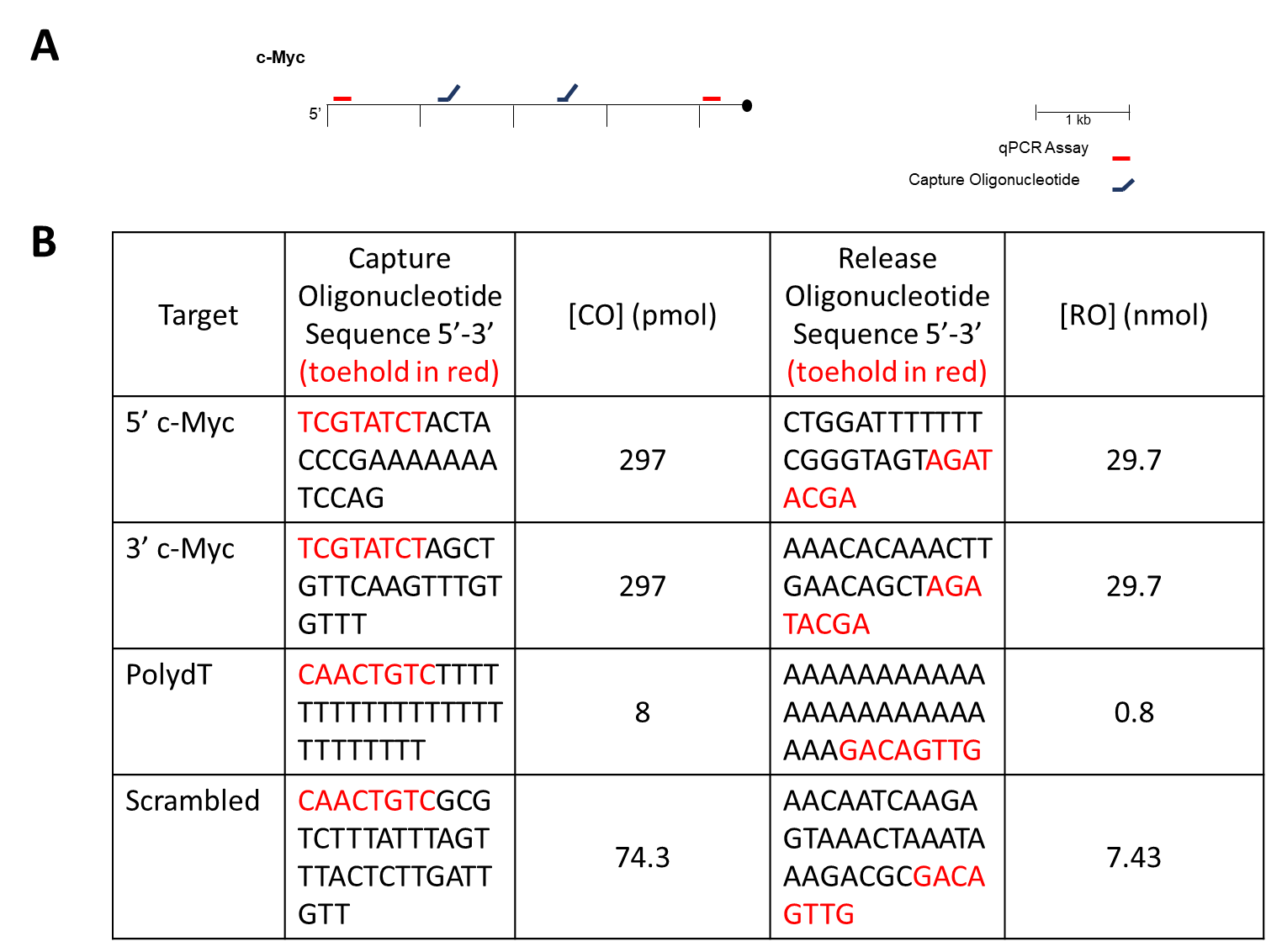


Figure S5: Oligonucleotide Sequences and Concentrations.

A) Two capture oligonucleotides were used to ensure uniform capture of the full c-Myc transcript length. To assess the number of c-Myc copies captured during method optimization, two qPCR assays were used at 3’ and 5’ ends of the transcript.

B) The sequences of the capture and release oligonucleotides, along with the concentrations of each, added to a large-scale HyPR-MS experiment.

C) The sequences of the qPCR primers and probes. The concentrations used were based on manufacturer instructions.

| eCLIP Target | eCLIP Experiment URL |
| --- | --- |
| AARS | https://www.encodeproject.org/experiments/ENCSR825SVO/ |
| AARS control | https://www.encodeproject.org/experiments/ENCSR696PRX/ |
| NOLC1 | https://www.encodeproject.org/experiments/ENCSR001VAC/ |
| NOLC1 control | https://www.encodeproject.org/experiments/ENCSR009CIV/ |
| YWHAG | https://www.encodeproject.org/experiments/ENCSR867ZVK/ |
| YWHAG control | https://www.encodeproject.org/experiments/ENCSR812PBU/ |
| IGF2BP1 | https://www.encodeproject.org/experiments/ENCSR975KIR/ |
| IGF2BP1 control | https://www.encodeproject.org/experiments/ENCSR125CLF/ |
| SND1 | https://www.encodeproject.org/experiments/ENCSR128VXC/ |
| SND1 control | https://www.encodeproject.org/experiments/ENCSR435EVK/ |
| SRSF1 | https://www.encodeproject.org/experiments/ENCSR432XUP/ |
| SRSF1 control | https://www.encodeproject.org/experiments/ENCSR925TYQ/ |

Figure S6: Publicly available eCLIP data used to validate AARS, IGFBP1, NOLC1, SND1, SRSF1, and YWHAG binding of c-Myc mRNA.
